# Psychosocial Adaptation in a Sensor-Augmented Telerehabilitation Program for Knee Osteoarthritis: A Post Hoc Analysis of a Randomized Controlled Trial

**DOI:** 10.3390/jcm15207924

**Published:** 2026-10-14

**Authors:** Theodora Plavoukou, Myrto Poulou, Amalia Continiero Spyropoulou, George Georgoudis

**Affiliations:** 1 Laboratory of Musculoskeletal Physiotherapy, Department of Physiotherapy, Faculty of Health and Caring Sciences, University of West Attica, Egaleo Campus, 12243 Athens, Greece; myrtopoulou@gmail.com (M.P.); amaaliacontiniero@uniwa.gr (A.C.S.); gg.physio@gmail.com (G.G.); 2 Department of Electrical and Electronic Engineering, University of West Attica, 12241 Egaleo, Greece

**Keywords:** self-efficacy, anxiety symptoms, depressive symptoms, kinesiophobia, wearable biofeedback, remote exercise, behavioral health, patient-reported outcomes

## Abstract

**Background**: Psychosocial adaptation during telerehabilitation for knee osteoarthritis (OA) is not well understood. Objective: To explore associations among self-efficacy, anxiety, depressive symptoms, and kinesiophobia during sensor-augmented telerehabilitation. **Methods**: This exploratory post hoc secondary analysis included 21 participants from the telerehabilitation arm of the KneE-PAD randomized controlled trial. Psychosocial outcomes were assessed before and after intervention using the Arthritis Self-Efficacy Scale (ASES), Hospital Anxiety and Depression Scale (HADS), and Tampa Scale for Kinesiophobia (TSK). Within-group changes were assessed using paired analyses with Holm–Bonferroni adjustment, associations among change scores were examined using correlation analyses with multiplicity correction, and exploratory regression models were fitted without multiplicity adjustment. **Results**: Improvement in pain-related self-efficacy was associated with reduced anxiety (ρ = −0.71, adjusted *p* = 0.006). No other correlation remained statistically significant after Holm–Bonferroni correction, and the regression findings were interpreted as exploratory. **Conclusions**: Psychosocial changes during sensor-augmented telerehabilitation were characterized by a strong association between improvement in pain-related self-efficacy and reduction in anxiety. These exploratory findings require confirmation in larger, prospectively designed studies. Trial Registration: ClinicalTrials.gov Identifier: NCT06416332.

## 1. Introduction

Knee osteoarthritis (OA) is a prevalent musculoskeletal disorder and a leading cause of pain, disability, and healthcare burden among older adults worldwide, currently affecting over 300 million individuals [1,2]. Beyond structural cartilage degeneration and inflammation, OA is increasingly recognized as a multidimensional condition involving neuromuscular impairments, proprioceptive deficits, and psychosocial comorbidities such as anxiety, fear of movement, and reduced self-efficacy, all of which negatively influence mobility and quality of life [3,4,5].

Exercise therapy remains the cornerstone of non-pharmacological OA management and consistently improves pain, physical function, and overall health outcomes across clinical settings [6,7,8,9,10,11]. Nevertheless, psychosocial factors substantially influence rehabilitation trajectories. Constructs such as self-efficacy, emotional distress, and fear of movement are closely linked to perceived disability and functional recovery [12,13,14,15]. Self-efficacy in particular has been identified as an important determinant of symptom management, behavioral engagement, and adaptation in people with arthritis [16,17].

Telerehabilitation has emerged as a scalable alternative to conventional physiotherapy, enabling remotely supervised exercise and continuity of rehabilitation care [13,18,19,20]. Systematic reviews in knee osteoarthritis indicate that digitally delivered and telerehabilitation-based exercise programs can improve pain and physical function, with outcomes in some settings comparable to conventional rehabilitation [13,15,20,21]. However, the psychological processes accompanying digital rehabilitation—particularly changes in self-efficacy, mood, and fear of movement—remain less clearly characterized.

Sensor-augmented telerehabilitation platforms can integrate wearable technologies such as surface electromyography (sEMG) and inertial measurement units (IMUs) to provide objective performance monitoring and real-time feedback during rehabilitation exercises [22,23,24,25]. In the primary KneE-PAD randomized controlled trial, sensor-augmented telerehabilitation demonstrated favorable neuromuscular, functional, and psychosocial outcomes in individuals with mild-to-moderate knee osteoarthritis [24]. However, the parent trial was designed primarily to evaluate the overall effectiveness of the intervention and did not specifically investigate the interrelationships among individual psychosocial changes. The present post hoc analysis therefore extends the findings of the parent trial by focusing specifically on within-group psychosocial adaptation and by examining associations among changes in self-efficacy, anxiety, depressive symptoms, and kinesiophobia. This focused analysis provides complementary, hypothesis-generating information regarding how psychosocial dimensions may co-evolve during sensor-augmented telerehabilitation, rather than simply re-reporting the clinical outcomes of the original trial.

Understanding these psychosocial adaptations is important within a biopsychosocial framework of rehabilitation. Improvements in self-efficacy may accompany symptom reduction and functional gains, and emotional changes may interact with fear of movement during recovery [16,17,26,27,28]. Exploring these interrelationships can inform the design of digitally supported rehabilitation systems that address not only motor performance but also psychological resilience.

Therefore, the present post hoc secondary analysis of the KneE-PAD randomized controlled trial [24] aimed to examine psychosocial changes within the telerehabilitation group and to explore associations among changes in self-efficacy (ASES subscales), anxiety and depressive symptoms (HADSs), and kinesiophobia (TSK). All analyses are explicitly exploratory and hypothesis-generating; findings are not confirmatory.

Accordingly, the following null hypotheses were examined:

**H0_1_.**  *Self-efficacy would not significantly change from baseline to post-intervention within the telerehabilitation group.*

**H0_2_.**  *Changes in self-efficacy would not be significantly associated with concurrent changes in anxiety, depressive symptoms, or kinesiophobia.*

## 2. Methodology

### 2.1. Study Design

This study represents a post hoc secondary analysis derived from the KneE-PAD randomized controlled trial (ClinicalTrials.gov Identifier: NCT06416332), which evaluated the effects of a sensor-augmented telerehabilitation program compared to conventional face-to-face physiotherapy in individuals with mild-to-moderate knee osteoarthritis (OA).

The parent trial employed a multicenter, single-blind, two-arm parallel design, with participants randomized to either telerehabilitation (*n* = 21) or in-person physiotherapy (*n* = 21). Both interventions were delivered over eight weeks, followed by a 12-week follow-up. Detailed methodology and primary neuromuscular and functional outcomes have been reported previously [24].

The present post hoc analysis focused exclusively on the telerehabilitation group (*n* = 21) and examined psychosocial adaptations occurring between baseline (T0) and post-intervention (T1). Specifically, associations among changes in self-efficacy (Arthritis Self-Efficacy Scale; ASES subscales), anxiety and depressive symptoms (Hospital Anxiety and Depression Scale; HADS), and kinesiophobia (Tampa Scale for Kinesiophobia; TSK) were explored. This analysis was exploratory and not pre-specified in the original trial protocol. All results are exploratory and should be used only to generate hypotheses for future confirmatory studies.

All analyses were conducted using observed data only. This secondary analysis adhered to CONSORT recommendations for transparent reporting of non-pharmacological randomized trials [29] and was conducted within the scope of the parent KneE-PAD trial [24].

### 2.2. Participants

#### 2.2.1. Sample Characteristics

Participants included in this post hoc analysis were those randomized to the telerehabilitation arm of the KneE-PAD trial (*n* = 21). All had radiographically confirmed mild-to-moderate knee osteoarthritis (Kellgren–Lawrence grade 1–3) and completed baseline (T0) and post-intervention (T1) psychosocial assessments. As previously reported in the parent trial [24], the mean age of participants in the telerehabilitation group was 57 ± 8 years, and 57% were female. Baseline demographic and clinical characteristics of the randomized groups have been reported previously [24].

#### 2.2.2. Inclusion and Exclusion Criteria

Participants were eligible if they were 40–70 years of age, had clinically and radiographically confirmed knee osteoarthritis corresponding to Kellgren–Lawrence grades 1–3, had sufficient literacy in Greek to understand the study procedures and exercise instructions, and had access to a stable internet connection required for the telerehabilitation sessions. Participants were excluded if they had an active psychiatric disorder or cognitive impairment that could interfere with completion of the self-report measures, or if they had recently participated in another behavioral or psychological intervention. All 21 participants allocated to the telerehabilitation arm met these criteria and had complete baseline and post-intervention psychosocial data.

#### 2.2.3. Ethical Considerations

All procedures were conducted in accordance with the Declaration of Helsinki [30] and were approved by the Research Ethics and Deontology Committee of the University of West Attica (approval no. 65417, approval date: 7 July 2023). Written informed consent was obtained from all participants prior to study participation, including consent for the scientific use and publication of anonymized data. Confidentiality was maintained throughout the secondary analysis.

### 2.3. Study Variables

#### 2.3.1. Psychosocial Outcomes

##### Self-Efficacy

Self-efficacy was assessed using the Arthritis Self-Efficacy Scale (ASES) [31], a validated instrument measuring confidence in managing arthritis-related symptoms. For the present analysis, the ASES Pain and ASES Symptoms subscales were examined. Each item is rated on a 10-point Likert scale ranging from 1 (“not confident”) to 10 (“very confident”), with higher scores indicating greater perceived self-efficacy. Change scores were calculated as the post-intervention score minus the baseline score (T1 − T0), such that positive values represented improvement in self-efficacy.

##### Anxiety and Depression

Anxiety and depressive symptoms were assessed using the Hospital Anxiety and Depression Scale (HADS) [32,33], comprising the HADS-A anxiety subscale and the HADS-D depression subscale. Each subscale ranges from 0 to 21, with higher scores reflecting greater symptom severity. Change scores were calculated as T1 − T0; therefore, negative values represented reductions in anxiety or depressive symptom severity.

##### Fear of Movement

Fear of movement was assessed using the Tampa Scale for Kinesiophobia (TSK) [26,34]. Total scores range from 17 to 68, with higher values indicating greater fear of movement. Change scores were calculated as T1 − T0, with negative values representing a reduction in kinesiophobia (Appendix A).

#### 2.3.2. Derived Change Variables

To evaluate within-group psychosocial change over the intervention period, difference scores were calculated for all outcomes as the post-intervention value minus the baseline value (T1 − T0). The derived variables included ΔASES Pain, ΔASES Symptoms, ΔHADS-A, ΔHADS-D, and ΔTSK. These change scores were subsequently used in the correlation and regression analyses to examine associations between changes in self-efficacy and concurrent changes in anxiety, depressive symptoms, and fear of movement. No adherence metrics or composite motivation indices were included in the dataset available for this secondary analysis. Accordingly, the present analyses were restricted to psychosocial outcomes and their change trajectories within the telerehabilitation arm.

### 2.4. Intervention Framework

Participants in the parent KneE-PAD randomized controlled trial were randomized to one of two intervention groups: (A) conventional in-person physiotherapy delivered in accordance with evidence-based recommendations for knee osteoarthritis, and (B) sensor-augmented telerehabilitation delivered through the KneE-PAD platform, which integrated surface electromyography (sEMG) and inertial measurement unit (IMU (Delsys Inc., Natick, MA, USA)), sensors to provide real-time visual and auditory feedback during exercise sessions [24,35]. Both interventions were delivered over an 8-week period, followed by a 12-week observational follow-up. The present post hoc analysis was restricted to participants allocated to the telerehabilitation arm.

The KneE-PAD intervention comprised structured exercise sessions based on progressive resistance and functional training principles. Real-time sensor-derived feedback enabled continuous monitoring of exercise performance and facilitated appropriate adjustments during each session. Remote supervision was provided by licensed physiotherapists to ensure consistency, safety, and adherence to the prescribed exercise protocol. In addition, the platform supported objective session tracking and performance monitoring throughout the intervention period; however, these platform-generated tracking data were not included in the dataset available for the present secondary analysis.

### 2.5. Data Collection Timeline

Psychosocial assessments were conducted at two predefined time points: baseline (T0), prior to intervention initiation, and post-intervention (T1), immediately following the 8-week program.

Analyses focused on change scores from T0 to T1 to capture short-term psychosocial adaptation during the intervention period.

### 2.6. Statistical Analysis

All statistical analyses were conducted using R software(version 4.3.1; R Foundation for Statistical Computing, Vienna, Austria). Statistical significance was set at *p* < 0.05 (two-tailed). Data distribution was assessed using the Shapiro–Wilk test. As change scores approximated normality, parametric methods were applied. Spearman correlation analyses were additionally performed as sensitivity checks to confirm robustness of directional associations. A detailed description of the statistical analysis workflow is provided in Appendix B.

#### 2.6.1. Descriptive Statistics and Power Limitations

Continuous variables are presented as mean ± standard deviation (SD). Categorical variables are reported as frequencies and percentages.

No a priori power calculation was conducted for this secondary analysis. The sample size (*n* = 21) was derived from the parent randomized controlled trial, which was powered for neuromuscular and functional outcomes, not psychosocial change. This sample size is insufficient to detect small-to-moderate effect sizes reliably and renders regression estimates potentially unstable. All findings are therefore exploratory. Within-group change scores were calculated as Δ = T1 − T0. For interpretative clarity, positive ΔASES values indicated improvement in self-efficacy, whereas negative ΔHADS and ΔTSK values indicated reductions in symptom severity and kinesiophobia, respectively.

Paired *t*-tests were used to evaluate within-group changes from baseline to post-intervention. Effect sizes for paired comparisons were calculated using Cohen’s d.

#### 2.6.2. Correlation Analysis

Associations between changes in psychosocial outcomes were examined using Pearson correlation coefficients. Spearman rank correlations were conducted as sensitivity analyses to verify the stability of observed relationships. The Holm–Bonferroni procedure was applied to control the family-wise error rate across the correlation analyses.

#### 2.6.3. Regression Models

Multiple linear regression models were constructed to examine associations between baseline self-efficacy and changes in emotional outcomes within the telerehabilitation group (*n* = 21). Given the exploratory and hypothesis-generating nature of the present post hoc analysis, all regression *p*-values are reported without correction for multiple comparisons. These *p*-values are therefore presented as descriptive measures of association strength and should not be interpreted as confirmatory evidence of statistical significance.

Because of the limited sample size, each regression model was restricted to a maximum of four predictors in order to reduce the risk of overfitting. The primary regression models examined whether baseline self-efficacy was associated with changes in depressive or anxiety symptoms after adjustment for the corresponding baseline symptom severity, age, and sex. An additional exploratory regression model examined whether change in depressive symptoms was associated with concurrent change in kinesiophobia. Change-score modeling was selected to evaluate within-individual psychosocial trajectories, while baseline outcome values were included where appropriate to reduce potential regression-to-the-mean bias.

For each model, standardized beta coefficients (β), R^2^, adjusted R^2^, and model F-statistics were reported. Given the modest sample size, R^2^ and adjusted R^2^ estimates were interpreted cautiously because of the potential for overestimation. Multicollinearity was assessed using variance inflation factors (VIFs). Model assumptions were evaluated through visual inspection of residual plots, including assessment of linearity, homoscedasticity, and normality of residuals.

Given the exploratory post hoc design and the limited sample size, all regression findings were interpreted as associative rather than causal. Accordingly, no confirmatory claims of statistical significance were made on the basis of uncorrected regression *p*-values.

#### 2.6.4. Effect Size and Multiple Testing

Effect sizes were quantified using Cohen’s d for paired within-group comparisons and standardized beta coefficients (β) for regression analyses. Model-level explained variance was summarized using R^2^ and adjusted R^2^ values. Given the modest sample size, these estimates were interpreted cautiously because of the potential for overestimation.

To account for the increased risk of type I error arising from multiple testing, the Holm–Bonferroni procedure was applied to the five paired within-group comparisons and to the correlation analyses. The three exploratory regression models were not adjusted for multiple comparisons. Accordingly, regression *p*-values are reported as uncorrected and are interpreted as exploratory and hypothesis-generating rather than confirmatory. No definitive confirmatory claims are made on the basis of these uncorrected regression results.

#### 2.6.5. Missing Data

All participants in the telerehabilitation group completed baseline and post-intervention psychosocial assessments. No imputation procedures were required, and analyses were performed using complete case data (Appendix C).

### 2.7. Use of Generative Artificial Intelligence in Manuscript Preparation

During manuscript preparation and revision, ChatGPT-6 (OpenAI) was used as an editorial support tool for language refinement, sentence restructuring, and improvement in the organization and readability of selected parts of the Abstract, Section 1, Section 2, Section 4 and Section 4.6. The tool was not used for study design, participant selection, data generation, statistical analysis, or autonomous interpretation of the findings. All statistical analyses were performed independently in R (version 4.3.1). All AI-assisted text was critically reviewed, verified against the original study data and cited literature, and edited by the authors, who take full responsibility for the final content of the manuscript.

## 3. Results

### 3.1. Sample Characteristics and Retention

The analysis included 21 participants (mean age 57 ± 8 years; 57% female). Retention was 100%, with complete data available for all psychosocial outcomes.

### 3.2. Psychosocial Outcomes (Telerehabilitation Group Only)

#### 3.2.1. Self-Efficacy (ASES Subscales)

Within the telerehabilitation group (*n* = 21), ASES-Symptoms increased from 5.96 ± 2.16 at baseline to 7.10 ± 1.91 post-intervention. The paired *t*-test yielded t(20) = −2.35, with an uncorrected *p* = 0.029 and a moderate effect size (Cohen’s d = −0.51). However, this change did not retain statistical significance after Holm–Bonferroni correction (adjusted *p* = 0.145). ASES-Pain increased from 6.50 ± 2.01 to 7.33 ± 1.69, but this change was not statistically significant (t(20) = −1.69, uncorrected *p* = 0.108; Cohen’s d = −0.37). Overall, the observed changes suggested small-to-moderate improvements in perceived self-efficacy, particularly for symptom management, but none of the within-group self-efficacy changes remained statistically significant after correction for multiple comparisons.

#### 3.2.2. Anxiety and Depression (HADS)

Depressive symptoms (HADS-D) showed minimal change from baseline (6.62 ± 3.68) to post-intervention (6.24 ± 3.71), with no statistically significant within-group difference (t(20) = 0.79, uncorrected *p* = 0.437; Cohen’s d = 0.17). Anxiety symptoms (HADS-A) decreased from 6.57 ± 3.87 to 5.24 ± 3.62, corresponding to a small effect size (Cohen’s d = 0.39). However, this change was not statistically significant after Holm–Bonferroni correction (t(20) = 1.78, uncorrected *p* = 0.091; adjusted *p* = 0.364). Overall, neither depressive nor anxiety symptoms demonstrated statistically significant within-group change after correction for multiple comparisons.

#### 3.2.3. Fear of Movement (TSK)

TSK scores decreased from 18.95 ± 5.98 at baseline to 16.62 ± 7.72 post-intervention, corresponding to a small reduction in kinesiophobia (Cohen’s d = 0.27). This change was not statistically significant (t(20) = 1.24, uncorrected *p* = 0.231) and did not retain statistical significance after correction for multiple comparisons (Table 1).

### 3.3. Correlation Analyses

Change-score associations were initially examined using Pearson correlations, and Spearman rank correlations were subsequently performed as sensitivity analyses, yielding directionally consistent findings. In the Spearman analysis, after Holm–Bonferroni correction for multiple comparisons, only the association between improvement in pain-related self-efficacy and reduction in anxiety remained statistically significant (ΔASES-Pain and ΔHADS-A: ρ = −0.71, raw *p* = 3.13 × 10^−4^; Holm-adjusted *p* = 0.006). The association between changes in depressive symptoms and kinesiophobia was moderate and positive (ΔHADS-D and ΔTSK: ρ = 0.60, raw *p* = 0.0037) but did not retain statistical significance after correction (Holm-adjusted *p* = 0.066) and was therefore interpreted as exploratory (Table 2). The complete Spearman correlation matrix is presented in Appendix D.

### 3.4. Regression Analyses

Multiple linear regression models were constructed to examine whether baseline self-efficacy was associated with change in emotional outcomes, adjusting for age and sex. All regression *p*-values are reported uncorrected for multiple comparisons. Given the exploratory nature of this analysis and the limited sample size, these *p*-values should not be interpreted as statistically significant in the confirmatory sense. No claims of causal relationships are made.

#### 3.4.1. Baseline ASES-Symptoms Predicting Depressive Change

A multiple linear regression model examined whether baseline ASES symptom-related self-efficacy was associated with change in depressive symptoms, adjusting for baseline depression severity, age, and sex. Baseline ASES-Symptoms was associated with change in depressive symptoms in the uncorrected analysis (β = 0.5536, *p* = 0.0467), whereas baseline HADS-D (β = 0.0132, *p* = 0.9308), age (β = −0.0856, *p* = 0.2003), and sex (β = −0.2964, *p* = 0.7808) were not associated with depressive change. The model explained 39.7% of the variance (R^2^ = 0.3966; adjusted R^2^ = 0.2457), although the overall F-test did not reach the conventional significance threshold (*p* = 0.073). Multicollinearity was minimal (all VIF < 1.7). Given that regression *p*-values were not adjusted for multiple comparisons and the sample size was limited, the observed association between baseline ASES-Symptoms and depressive change should be interpreted as exploratory and hypothesis-generating.

#### 3.4.2. Baseline ASES-Pain Predicting Anxiety Change

A multiple linear regression model examined whether baseline pain-related self-efficacy was associated with change in anxiety symptoms, adjusting for baseline anxiety, age, and sex. Neither ASES_PAIN_Total (β = 0.60, *p* = 0.195) nor HADS_A_Total (β = −0.37, *p* = 0.136) were statistically significant in uncorrected analyses, and age (uncorrected *p* = 0.164) and gender (uncorrected *p* = 0.237) were also not significant. The model explained 39.8% of the variance (R^2^ = 0.398; adjusted R^2^ = 0.248), but did not reach statistical significance (overall F-test *p* = 0.072). Variance inflation factors were below 2 for all predictors, suggesting no evidence of multicollinearity. Although a strong correlation was observed between changes in pain self-efficacy and anxiety (Section 3.3), baseline pain self-efficacy was not significantly associated with anxiety improvement after adjustment for covariates in uncorrected analyses.

#### 3.4.3. Association Between Changes in Depression and Kinesiophobia

To examine whether changes in depressive symptoms co-occurred with changes in fear of movement, a linear regression model assessed the association between change in depressive symptoms and change in kinesiophobia, adjusting for age and sex. Change in depressive symptoms was associated with change in kinesiophobia in the uncorrected analysis (β = 2.444, *p* = 0.0069), whereas age (β ≈ 0.000007, *p* = 0.99998) and sex (β = −0.0559, *p* = 0.9890) were not associated with kinesiophobia change. The model explained 38.8% of the variance (R^2^ = 0.3882; adjusted R^2^ = 0.2802), and the overall F-test yielded an uncorrected *p* = 0.035. Variance inflation factors were below 2 for all predictors (VIF < 1.6), indicating no evidence of multicollinearity. Given the modest sample size and the absence of multiplicity correction for the regression models, these findings should be interpreted as exploratory and hypothesis-generating rather than confirmatory. No causal or directional relationship can be inferred from this association.

## 4. Discussion

### 4.1. Psychosocial Adaptation Within Telerehabilitation

This exploratory secondary analysis extends the findings of the parent KneE-PAD randomized controlled trial [24], which primarily evaluated the overall neuromuscular, functional, and psychosocial effects of sensor-augmented telerehabilitation. In contrast, the present analysis specifically examined how psychosocial changes were interrelated within the telerehabilitation group, focusing on change-score associations among self-efficacy, anxiety, depressive symptoms, and kinesiophobia. After correction for multiple comparisons, only the association between improvement in pain-related self-efficacy and reduction in anxiety remained statistically significant. Within-group changes in self-efficacy, mood, and fear of movement did not retain statistical significance after correction. Thus, the novel contribution of the present study lies not in re-evaluating the overall effectiveness of the intervention, but in exploring the pattern of psychosocial adaptation occurring within the digitally supported rehabilitation context. These findings are hypothesis-generating and require confirmation in larger, prospectively designed studies.

At the review level, evidence supporting digitally delivered rehabilitation is encouraging but remains heterogeneous. Systematic reviews of musculoskeletal telerehabilitation and internet-based rehabilitation have generally reported improvements in pain and physical function, with outcomes in some settings comparable to conventional care; however, substantial variation in technological platforms, supervision, intervention intensity, and outcome selection limits conclusions regarding the specific mechanisms responsible for these benefits [13,15,21]. More recent evidence further supports the clinical relevance of digital rehabilitation for knee osteoarthritis. A 2025 systematic review of telerehabilitation reported beneficial effects on pain, strength, and balance, while emphasizing heterogeneity in intervention protocols, delivery methods, and patient engagement [36]. Similarly, a 2025 systematic review and meta-analysis found moderate-quality evidence that digital physiotherapy produced a small improvement in physical function compared to standard physiotherapy, while considerable heterogeneity limited quantitative conclusions for pain and quality of life [37]. Psychosocial outcomes, however, remain less consistently assessed and comparatively underexplored. In a broader mental-health context, Petre et al. [38] systematically reviewed internet- and mobile-based aftercare interventions for anxiety and depressive disorders and reported generally favorable effects, particularly for depressive symptoms, while also highlighting variability in adherence, intervention characteristics, and methodological quality. Although the populations and therapeutic objectives examined in that review differ from those of the present knee osteoarthritis cohort, the findings reinforce an important broader principle: digital delivery alone should not be assumed to produce psychosocial benefit. Rather, factors such as engagement, professional support, personalization, and intervention design may influence psychological outcomes. Accordingly, the association observed in the present study between improvement in pain-related self-efficacy and reduction in anxiety should be interpreted as hypothesis-generating rather than as evidence that sensor-augmented telerehabilitation directly modifies psychological mechanisms.

Associations between self-efficacy and emotional outcomes were observed in the present analysis, although their direction and statistical robustness varied across models. Self-efficacy has been shown to influence coping, adherence, and symptom perception in musculoskeletal conditions [7,39], supporting its role as a central psychological resource during rehabilitation. In the present study, improvements in pain-related self-efficacy were associated with reductions in anxiety, reinforcing theoretical models suggesting that perceived control over symptoms may mitigate emotional distress [16,17].

Furthermore, the observed association between changes in depressive symptoms and kinesiophobia did not retain statistical significance after Holm–Bonferroni correction and should therefore be interpreted as exploratory. This pattern is nevertheless compatible with literature describing relationships between affective distress, fear-related responses, and functional outcomes in knee osteoarthritis [27,28].

Importantly, these findings reflect concurrent associations and do not establish causal or temporal relationships among psychosocial variables. Although real-time feedback and structured supervision are features of sensor-augmented telerehabilitation that may support perceived competence and engagement, the present study did not directly test these mechanisms [13,23,40].

### 4.2. Self-Efficacy and Emotional Change

Self-efficacy is widely recognized as an important psychological construct in musculoskeletal rehabilitation [16,17]. In the present analysis, improvement in pain-related self-efficacy was strongly associated with reduction in anxiety (ρ = −0.71, Holm-adjusted *p* = 0.006). Baseline symptom-related self-efficacy was also associated with change in depressive symptoms in the uncorrected regression analysis (β = 0.55, *p* = 0.047). However, regression *p*-values were not adjusted for multiple comparisons, and the overall regression model did not reach the conventional threshold for statistical significance (*p* = 0.073). This finding should therefore be interpreted as exploratory and hypothesis-generating rather than confirmatory.

Although depressive symptoms did not demonstrate a statistically significant mean-level change, baseline symptom-related self-efficacy was associated with change in depressive symptoms in the exploratory regression model. Given the coding of change scores and the positive regression coefficient, this finding should not be interpreted as indicating that higher baseline self-efficacy predicted greater improvement in depressive symptoms. Moreover, the modest sample size, unadjusted regression *p*-value, and non-significant overall model warrant cautious interpretation.

The association between improvements in pain-related self-efficacy and reductions in anxiety further supports the potential relevance of perceived symptom control to emotional responses during rehabilitation. Previous work has suggested that self-efficacy may influence how individuals interpret and respond to pain-related experiences, with greater perceived control being associated with more adaptive coping and lower emotional distress [16,17]. In knee osteoarthritis, where symptoms may fluctuate over time, greater confidence in managing pain may therefore be linked to reduced anxiety and threat-related responses.

Within digitally delivered rehabilitation, real-time performance feedback and structured professional supervision may provide repeated opportunities for mastery experiences and reinforce confidence in movement and symptom management. Such mechanisms may contribute to the concurrent improvements observed in self-efficacy and anxiety; however, the present study was not designed to test mediation or underlying psychological mechanisms directly. Accordingly, these findings should be interpreted as evidence of association rather than causation.

Importantly, all observed relationships should be considered within the exploratory post hoc framework of the present analysis. Larger, prospectively designed studies with predefined psychosocial outcomes are required to confirm these associations and to determine whether changes in self-efficacy contribute meaningfully to emotional adaptation during telerehabilitation.

### 4.3. Interrelationship Between Depression and Kinesiophobia

A moderate association was observed between changes in depressive symptoms and changes in kinesiophobia in uncorrected analyses (ρ = 0.60, uncorrected *p* = 0.0037), indicating that participants who experienced greater reductions in depressive symptoms also demonstrated greater reductions in fear of movement. However, this association did not retain statistical significance after Holm–Bonferroni correction (adjusted *p* = 0.066) and is therefore considered an exploratory finding requiring confirmation. The absence of temporal separation between measurements precludes inference regarding directionality.

This exploratory association is consistent with a biopsychosocial interpretation of knee osteoarthritis, in which emotional distress and fear-related responses may interact and contribute to avoidance behavior and functional limitation [27,28]. Negative affective states may amplify pain-related threat perception and movement-related fear, although the present analysis cannot establish the direction of these relationships [28].

Within a biopsychosocial framework, affective distress, self-efficacy, and fear-related responses may interact during rehabilitation rather than operate as isolated constructs [27,28]. In knee osteoarthritis, these psychosocial factors have been associated with functional status and rehabilitation-related outcomes, supporting their consideration as interrelated components of the patient experience [27,28].

Future research should prospectively examine whether the intensity, frequency, or personalization of real-time feedback influences psychosocial adaptation during telerehabilitation. Comparative designs contrasting sensor-based interactive systems with minimally supervised or asynchronous digital exercise programs may help clarify whether feedback acts as an active mechanism of psychological change or primarily reflects participant engagement. Longitudinal designs incorporating intermediate assessment points would further enable evaluation of whether changes in emotional state and fear of movement evolve sequentially or concurrently.

### 4.4. Clinical Implications

The present findings highlight the potential clinical value of integrating psychosocial monitoring within digitally delivered rehabilitation programs. The implications discussed are speculative and derived from exploratory, hypothesis-generating analyses. Therefore, they should not be interpreted as practice recommendations without confirmation in larger, prospectively designed studies.

If confirmed in future studies, the observed associations would suggest that incorporating psychosocial monitoring—particularly of self-efficacy and anxiety—into digital rehabilitation platforms might be worthy of investigation. However, at present, no clinical recommendations can be made based on these exploratory findings.

### 4.5. Limitations and Future Directions

Several limitations should be acknowledged. First, the modest sample size of the telerehabilitation subgroup (*n* = 21) limits statistical power and generalizability and may increase the instability of regression estimates. No a priori power calculation was conducted for this secondary analysis, as the sample size was determined by the parent randomized controlled trial, which was powered for neuromuscular and functional outcomes. The regression models explained a modest-to-moderate proportion of variance, with adjusted R^2^ values of approximately 0.25–0.28. Given the exploratory post hoc design and the psychosocial nature of the outcomes, these estimates were interpreted descriptively rather than as measures of predictive performance. Greater emphasis was therefore placed on the direction, consistency, and theoretical coherence of the observed associations, while avoiding confirmatory or causal interpretation.

Second, this investigation represents a post hoc secondary analysis that was not pre-registered in the original randomized controlled trial protocol. As such, the findings are hypothesis-generating and were not derived from a study specifically designed to examine psychosocial mechanisms.

Third, psychosocial outcomes were assessed at only two time points, baseline and post-intervention. This design does not provide sufficient temporal separation between potential predictors, mediators, and outcomes to support formal mediation analysis. Because self-efficacy, anxiety, depressive symptoms, and kinesiophobia were assessed concurrently at each time point, temporal precedence cannot be established. Accordingly, the observed relationships should be interpreted as concurrent associations rather than evidence of mediational or causal pathways. Future studies incorporating multiple intermediate assessment points and larger samples would be required to evaluate potential mediation mechanisms.

Fourth, the analysis focused exclusively on the telerehabilitation arm to examine within-context psychosocial dynamics. Consequently, direct between-group comparisons were not performed, and findings should be interpreted within a single-group exploratory framework [29].

Fifth, no correction for multiple comparisons was applied to the regression models; therefore, the uncorrected *p*-values reported (including *p* = 0.047 and *p* = 0.0069) are at elevated risk of false-positive findings and should not be interpreted as statistically significant in the confirmatory sense.

Sixth, this exploratory analysis was not designed to evaluate clinically meaningful change, and intervention-specific minimal clinically important difference thresholds were not applied [41,42,43,44]. Accordingly, the observed score changes and effect sizes should not be interpreted as evidence of clinical meaningfulness.

Future research should include larger, prospectively designed and adequately powered trials with pre-registered psychosocial endpoints and longitudinal designs capable of capturing temporal relationships among self-efficacy, emotional adaptation, and fear of movement. Such approaches are recommended in contemporary guidelines for osteoarthritis research and rehabilitation trials [45,46]. Comparative studies examining different telerehabilitation delivery models, including variations in feedback intensity and personalization, may further clarify the mechanisms underlying psychosocial adaptation in digitally delivered rehabilitation [13,15,21].

### 4.6. Conclusions

In this exploratory, hypothesis-generating post hoc analysis of a sensor-augmented telerehabilitation program for knee osteoarthritis, improvement in pain-related self-efficacy was strongly associated with reduction in anxiety (ρ = −0.71, Holm-adjusted *p* = 0.006). None of the within-group changes in self-efficacy, anxiety, depressive symptoms, or kinesiophobia retained statistical significance after correction for multiple comparisons. Baseline symptom-related self-efficacy was associated with change in depressive symptoms in an uncorrected regression analysis (β = 0.55, *p* = 0.047); however, the overall regression model did not reach the conventional significance threshold, and this finding should therefore be interpreted as exploratory. These findings are consistent with a biopsychosocial perspective on digitally delivered physiotherapy but require confirmation in larger, prospectively designed and adequately powered studies with predefined psychosocial outcomes. No causal or definitive clinical conclusions can be drawn from the present data.

## Figures and Tables

**Table 1:** Within-Group Psychosocial Changes (Telerehabilitation, *n* = 21).

Measure	T0 Mean ± SD	T1 Mean ± SD	t (df = 20)	Raw *p*	Holm-Adjusted *p*	Cohen’s d
ASES-Symptoms	5.96 ± 2.16	7.10 ± 1.91	−2.35	0.029	0.145	−0.51
ASES-Pain	6.50 ± 2.01	7.33 ± 1.69	−1.69	0.108	0.364	−0.37
HADS-D	6.62 ± 3.68	6.24 ± 3.71	0.79	0.437	0.462	0.17
HADS-A	6.57 ± 3.87	5.24 ± 3.62	1.78	0.091	0.364	0.39
TSK	18.95 ± 5.98	16.62 ± 7.72	1.24	0.231	0.462	0.27

Note. Holm–Bonferroni correction was applied across the five paired within-group comparisons. None of the within-group changes retained statistical significance after correction for multiple comparisons. Negative Cohen’s d values for ASES reflect improvement because higher ASES scores indicate greater self-efficacy.

**Table 2:** Correlations Among Change Scores.

Variable 1	Variable 2	ρ	*p* (Raw)	Holm-Adjusted *p*
ΔASES-Pain	ΔHADS-A	−0.71	3.13 × 10^−4^	0.006
ΔHADS-D	ΔTSK	0.60	0.0037	0.066

Note. Spearman rank correlations are reported. Holm–Bonferroni correction was applied across the correlation analyses. Only the association between ΔASES-Pain and ΔHADS-A retained statistical significance after correction for multiple comparisons; the association between ΔHADS-D and ΔTSK is considered exploratory.

## Data Availability

The publicly available KneE-PAD dataset comprises sensor-based knee rehabilitation exercise data and is accessible on Zenodo (https://doi.org/10.5281/zenodo.12112951 [47]). The psychosocial data analyzed in the present post hoc study are not included in the public dataset. De-identified data supporting the findings of this secondary analysis and the corresponding R analysis scripts may be available from the corresponding author upon reasonable request, subject to applicable ethical and data-protection requirements.

## Abbreviations

ASES	Arthritis Self-Efficacy Scale
HADS	Hospital Anxiety and Depression Scale
TSK	Tampa Scale for Kinesiophobia
MCID	Minimal Clinically Important Difference
RCT	Randomized Controlled Trial
KneE-PAD	Knee Exercise—Postural Assessment Dataset

## Appendix A. Psychometric Instruments and Scoring

The following validated instruments were used to assess psychosocial constructs during the KneE-PAD telerehabilitation program.

**Instrument**	**Construct**	**No. of Items**	**Response Scale**	**Score Range**	**Cronbach’s α**	**Reference**
**ASES**	Arthritis Self-Efficacy (pain, function, symptoms)	20	1–10 Likert	1–10	0.88	Lorig et al., 1989 [16]; Christakou et al., 2024 [31]
**HADS**	Anxiety and Depression	14	0–3 Likert	0–21 per subscale	0.83	Zigmond and Snaith, 1983 [33]; Michopoulos et al., 2008 [32]
**TSK**	Fear of Movement (Kinesiophobia)	17	1–4 Likert	17–68	0.84	Monticone et al., 2017 [48]; Georgoudis et al., 2005 [34]; Georgoudis et al., 2022 [26]
All questionnaires were administered in validated Greek versions.						

## Appendix B. Statistical Analysis Workflow

**Software**

All statistical analyses were conducted using R software (version 4.3.1).

**Data Validation and Assumption Testing**

Data were screened for completeness, range errors, and potential outliers prior to statistical analysis. Normality of continuous variables and change scores was assessed using the Shapiro–Wilk test. For the regression models, diagnostic plots were inspected to assess linearity, homoscedasticity, and normality of residuals. Multicollinearity was evaluated using variance inflation factors (VIFs), with values below 3 considered acceptable.

**Main Analyses**
**Within-group changes (T0 → T1):**

Paired *t*-tests were used to evaluate changes in psychosocial outcomes from baseline (T0) to post-intervention (T1). Cohen’s d was calculated to quantify the magnitude of within-group change. The Holm–Bonferroni procedure was applied to the paired within-group comparisons to account for multiple testing.

**Correlation analyses:**

Pearson correlation coefficients were calculated to examine associations among change scores in ASES Pain, ASES Symptoms, HADS-A, HADS-D, and TSK. Spearman rank correlations were additionally performed as sensitivity analyses to assess the robustness and direction of the observed associations. The Holm–Bonferroni procedure was applied to the correlation analyses to control the family-wise error rate arising from multiple testing.

**Regression analyses:**

Multiple linear regression models were constructed to examine associations between baseline self-efficacy and changes in emotional outcomes within the telerehabilitation group. Models examining change in anxiety or depressive symptoms were adjusted for the corresponding baseline symptom severity, age, and sex. An additional exploratory model examined the association between change in depressive symptoms and concurrent change in kinesiophobia, adjusting for age and sex. Given the limited sample size, each model was restricted to a maximum of four predictors. Standardized beta coefficients (β), R^2^, adjusted R^2^, and overall model statistics were reported.

Regression *p*-values were not adjusted for multiple comparisons. Accordingly, all regression findings were interpreted as exploratory and hypothesis-generating rather than confirmatory, and no causal interpretation was made.

**Significance Threshold**

Statistical significance was set at *p* < 0.05 (two-tailed). Holm–Bonferroni correction was applied to the paired within-group comparisons and correlation analyses. Regression *p*-values were reported without multiplicity correction and were interpreted cautiously within the exploratory post hoc framework.

**Missing Data**

All participants in the telerehabilitation subgroup had complete baseline and post-intervention psychosocial data. Therefore, no imputation procedures were required, and all analyses were performed using complete-case data.

## Appendix C. Data Verification and Integrity

All psychosocial variables (ASES, HADS, TSK) were extracted directly from the validated dataset of the parent randomized controlled trial. Data integrity had been previously verified during the primary trial through double data entry checks and consistency screening procedures. For the present post hoc analysis, datasets were screened for completeness, range errors, and outliers prior to statistical modeling. No missing values were identified within the telerehabilitation subgroup for T0 and T1 assessments. Regression assumptions were examined through residual diagnostics and multicollinearity testing (VIF). Visual inspection of the diagnostic plots did not indicate major violations of the assessed model assumptions; however, regression estimates were interpreted cautiously given the limited sample size.

## Appendix D. Correlation Matrix of Key Variables

**Variable**	**ΔASES Pain**	**ΔASES Symptoms**	**ΔHADS-A**	**ΔHADS-D**	**ΔTSK**
ΔASES Pain	1.00	0.58	−0.71 **	−0.49	−0.18
ΔASES Symptoms		1.00	−0.42	−0.49	−0.20
ΔHADS-A			1.00	0.51	0.22
ΔHADS-D				1.00	0.60
ΔTSK					1.00
Note. Values are Spearman rank correlation coefficients (ρ) among change scores. Holm–Bonferroni correction was applied across the correlation analyses. ** *p* < 0.01 indicates statistical significance after Holm adjustment.					
